# Different association of atherogenic index of plasma with the risk of high platelet reactivity according to the presentation of acute myocardial infarction

**DOI:** 10.1038/s41598-024-60999-3

**Published:** 2024-05-13

**Authors:** Ki-Bum Won, Hyeon Jeong Kim, Jun Hwan Cho, Sang Yup Lee, Ae-Young Her, Byeong-Keuk Kim, Hyung Joon Joo, Yongwhi Park, Kiyuk Chang, Young Bin Song, Sung Gyun Ahn, Jung-Won Suh, Jung Rae Cho, Hyo-Soo Kim, Moo Hyun Kim, Do-Sun Lim, Sang-Wook Kim, Young-Hoon Jeong, Eun-Seok Shin

**Affiliations:** 1 Division of Cardiology, Chung-Ang University Gwangmyeong Medical Center, Chung-Ang University College of Medicine, Gwangmyeong, South Korea; 2 Division of Cardiology, Busan Veterance Hospital, Busan, South Korea; 3 Division of Cardiology, Kangwon National University School of Medicine, Chuncheon, South Korea; 4 Division of Cardiology, Severance Cardiovascular Hospital, Yonsei University College of Medicine, Seoul, South Korea; 5 Division of Cardiology, Cardiovascular Center, Korea University Anam Hospital, Korea University College of Medicine, Seoul, South Korea; 6 Division of Cardiology, Gyeongsang National University Changwon Hospital, Gyeongsang National University School of Medicine, Changwon, South Korea; 7 Division of Cardiology, Seoul St. Mary’s Hospital, College of Medicine, Catholic University of Korea, Seoul, South Korea; 8 Division of Cardiology, Samsung Medical Center, Sungkyunkwan University School of Medicine, Seoul, South Korea; 9 Division of Cardiology, Yonsei University Wonju Severance Christian Hospital, Wonju, South Korea; 10 Division of Cardiology, Seoul National University Bundang Hospital, Seongnam, South Korea; 11 Division of Cardiology, Kangnam Sacred Heart Hospital, Hallym University College of Medicine, Seoul, South Korea; 12 Division of Cardiology, Seoul National University Hospital, Seoul, South Korea; 13 Division of Cardiology, Dong-A University Hospital, Busan, South Korea; 14 Division of Cardiology, Ulsan University Hospital, University of Ulsan College of Medicine, 877 Bangeojinsunhwando-ro, Dong-gu, Ulsan, 44033 South Korea

**Keywords:** Plasma atherogenicity, Platelet reactivity, Acute myocardial infarction, Prognosis, Percutaneous coronary intervention, Drug-eluting stents, Biomarkers, Cardiology, Acute coronary syndromes

## Abstract

This study evaluated the association of atherogenic index of plasma (AIP) with platelet reactivity and clinical outcomes according to acute myocardial infarction (AMI). The composite of 3-year adverse outcomes of all-cause death, myocardial infarction, and cerebrovascular accident was evaluated in 10,735 patients after successful percutaneous coronary intervention with drug-eluting stents. AIP was defined as the base 10 logarithm of the ratio of triglyceride to high-density lipoprotein cholesterol concentration. High platelet reactivity (HPR) was defined as ≥ 252 P2Y12 reactivity unit. An increase of AIP (per-0.1 unit) was related to the decreased risk of HPR [odds ratio (OR) 0.97, 95% confidence interval (CI) 0.96–0.99; P = 0.001] in non-AMI patients, not in AMI patients (OR 0.98, 95% CI 0.96–1.01; P = 0.138). The HPR was associated with the increased risk of composite outcomes in both non-AMI and AMI patients (all-P < 0.05). AIP levels were not independently associated with the risk of composite outcomes in both patients with non-AMI and AMI. In conclusion, an inverse association between AIP and the risk of HPR was observed in patients with non-AMI. This suggests that the association between plasma atherogenicity and platelet reactivity may play a substantial role in the development of AMI.

Trial registration: NCT04734028.

## Introduction

Atherosclerosis and its related cardiovascular (CV) diseases are the leading causes of mortality and major contributors to disability worldwide^1,2^. The atherogenic lipoprotein profile of plasma is one of the most important risk factors for atherosclerosis. The atherogenic index of plasma (AIP), which is based on the ratio of triglyceride to high-density lipoprotein (HDL) cholesterol concentrations, has been suggested as a marker of plasma atherogenicity because of its strong and positive relationship with cholesterol esterification rates, lipoprotein particle size, and remnant lipoproteinemia^3,4^. According to the recent data from the PARADIGM (The Progression of Atherosclerotic Plaque Determined by Computed Tomography Angiography Imaging) registry, high AIP levels were independently associated with an increased risk of rapid progression of coronary atherosclerosis beyond the traditional risk factors among adults with low to intermediate CV risk^5^. However, the association between plasma atherogenicity with platelet reactivity remains unclear. Considering that high platelet reactivity (HPR) is an independent predictor of adverse ischemic events after percutaneous coronary intervention (PCI) using drug-eluting stents (DES)^6–8^, this might be a substantial issue in clinical practice. Additionally, there is a paucity of data on the prognostic significance of AIP in the recent era of PCI with DES. Based on the evidence of an explicitly different pathogenesis according to the event of acute myocardial infarction (AMI)^9,10^, the present study aimed to investigate 1) the association of plasma atherogenicity assessed by AIP with the risk of HPR and 2) the prognostic value of AIP among patients who were successfully treated using PCI with DES according to the presentation of AMI.

## Methods

### Study design and population

This study analyzed the data of the PTRG-DES (the Platelet function and genotype-Related long-term prognosis in Drug-Eluting Stent–treated patients with coronary artery disease) consortium consisting of 13,160 patients who underwent successful PCI with DES for obstructive coronary artery disease (CAD) in South Korea between July 2003 and August 2018^11^. All patients underwent PCI with at least one DES and received dual antiplatelet therapy (DAPT) with clopidogrel and aspirin. This multicenter cohort study enrolled 10,735 patients based on the following criteria: (a) VerifyNow P2Y12 test during clopidogrel treatment, (b) no plan to undergo bypass surgery after the index PCI, (c) absence of major complications before the platelet function test, (d) no use of oral anticoagulants or P2Y12 inhibitors other than clopidogrel, and (e) available AIP data.

All participants were assessed for the requirement of loading doses of DAPT at the time of index PCI; accordingly, 300 mg aspirin and 300–600 mg clopidogrel were administered prior to the PCI procedure. Maintenance of DAPT was recommended for 12 months; however, discontinuation of DAPT was left to the discretion of each physician. Baseline and on-treatment clopidogrel platelet reactivity was measured using the VerifyNow P2Y12 point-of-care assay (Accumetrics, San Diego, CA, USA). The results of the platelet function tests were presented as VerifyNow P2Y12 reaction units (PRU). HPR was defined as a PRU of > 252 based on previous studies involving East Asians^12^. AIP was calculated as the base 10 logarithm of the ratio of triglyceride to HDL cholesterol concentrations^3,4^. A high AIP level was defined as an AIP of more than 0.54 based on a triglyceride/HDL cholesterol cutoff point of 3.5^13,14^. Body mass index (BMI) was calculated as weight in kilograms divided by the square of the height in meters (kg/m^2^). Clinical follow-up was performed either via a visit to the outpatient clinic or by telephone interview with the patient at the end of the first month and every 3 or 6 months after the PCI procedure. Informed consent for procedures was obtained from all participants at each of centers. All methods were performed following relevant guidelines and regulations and this study was performed in accordance with the Good Clinical Practice Guidelines and principles of the Declaration of Helsinki. The study was approved by the Institutional Review Board of the Ulsan University Hospital.

The primary endpoint of this study was 3-year composite events, including all-cause death, myocardial infarction (MI), or cerebrovascular accident (CVA), after PCI with DES. MI was defined as the presence of clinical symptoms, electrocardiographic changes, or abnormal imaging findings associated with MI, combined with an increase in the creatine kinase-myocardial band above the upper normal limit, or troponin I/T above the 99th percentile of the upper normal limit, unrelated to an interventional procedure^15^. CVA was defined as any new event of embolic, thrombotic, or hemorrhagic stroke with neurological deficits that persisted for at least 24 h. Major bleeding was defined as Bleeding Academic Research Consortium type ≥ 3.

### Statistical analysis

Continuous variables were presented as the mean ± standard deviation and categorical variables were presented as the absolute number (percentage). To compare the characteristics between groups, we employed an independent t-test, Mann–Whitney U test, one-way analysis of variance, or Kruskal–Wallis test for continuous variables; and the chi-squared test or Fisher’s exact test for categorical variables, as appropriate. The restricted cubic spline analysis for the association between AIP and the risk of HPR was performed according to the presentation of AMI. Odds ratios (OR) and 95% confidence interval (CI) were calculated using logistic regression. The cumulative incidence of adverse clinical events was estimated using the Kaplan–Meier method. Hazard ratios (HR) and 95% CI were calculated using Cox proportional hazard models. The forced entry method was used to enter the independent variables into the multiple logistic and Cox proportional hazards regression models. C statics, net reclassification index, and integrated discrimination index were calculated to evaluate an additive predictive value of AIP beyond HPR, clinical risk factor, and heart failure. All statistical analyses were performed using R (version 3.6.3; R Foundation for Statistical Computing, Vienna, Austria). Statistical significance was set at P < 0.05 for all analyses.

## Results

### Baseline characteristics

The baseline characteristics of the study participants are presented in Table 1. The mean age of the 10,735 patients (7252 male, 67.6%) was 64.4 ± 10.9 years. AMI was observed in 29.2% of the patients. Compared with non-AMI patients, the proportion of age ≥ 75 years, smoking, chronic kidney disease, peripheral artery disease, multivessel disease, the levels of total cholesterol and low-density lipoprotein (LDL) cholesterol, and the medications at discharge including beta-blocker, angiotensin blockade, and statin were significantly higher in patients with AMI. Patients without AMI had a higher left ventricular ejection fraction (LVEF) than those with AMI. There were no significant differences in PRU levels or the proportion of HPR between patients with and without AMI. However, the levels of AIP (0.46 ± 0.29 vs. 0.44 ± 0.30; P < 0.001) and the proportion of high AIP (37.9% vs. 35.2%; P = 0.008) were higher in patients with non-AMI than in those with AMI. Baseline characteristics related to HPR and AIP status in non-AMI and AMI patients are described in Supplementary Table 1.

**Table 1:** Baseline characteristics.

	Total (n = 10,735)	Non-AMI (n = 7599)	AMI (n = 3136)	P
Age, years	64.4 ± 10.9	64.5 ± 10.2	64.1 ± 12.4	0.160
Age ≥ 75 years	2030 (18.9)	1313 (17.3)	717 (22.9)	< 0.001
Male	7252 (67.6)	5060 (66.6)	2192 (69.9)	0.001
BMI, kg/m^2^	24.6 ± 3.1	24.8 ± 3.1	24.0 ± 3.2	< 0.001
LVEF, %	58.8 ± 10.6	60.9 ± 9.9	54.4 ± 10.5	< 0.001
Previous medical history				
Hypertension	6477 (60.3)	4825 (63.5)	1652 (52.7)	< 0.001
Diabetes mellitus	3714 (34.6)	2772 (36.5)	942 (30.0)	< 0.001
Dyslipidemia	7254 (67.6)	5111 (67.3)	2143 (68.3)	0.279
Obesity	4550 (42.4)	3452 (45.4)	1098 (35.0)	< 0.001
Smoking	3021 (28.1)	1791 (23.6)	1230 (39.2)	< 0.001
Chronic kidney disease	2224 (20.7)	1509 (19.9)	715 (22.8)	0.001
Peripheral artery disease	1305 (12.2)	802 (10.6)	503 (16.0)	< 0.001
Previous PCI	1445 (13.5)	1151 (15.1)	294 (9.4)	< 0.001
Previous CVA	745 (6.9)	526 (6.9)	219 (7.0)	0.909
Procedural data				
Multivessel disease	4244 (39.5)	2812 (37.0)	1432 (45.7)	< 0.001
Bifurcation lesion	1258 (11.7)	868 (11.4)	390 (12.4)	0.138
CTO lesion	747 (7.0)	557 (7.3)	190 (6.1)	0.019
Number of stents	1.6 ± 0.8	1.6 ± 0.8	1.6 ± 0.8	0.938
Length of stent, mm	35.9 ± 22.7	35.7 ± 23.1	36.3 ± 21.5	0.212
Minimal diameter of stent, mm	3.02 ± 0.44	3.00 ± 0.43	3.06 ± 0.47	< 0.001
Laboratory data				
Hemoglobin, g/dL	13.6 ± 1.8	13.6 ± 1.7	13.7 ± 2.0	< 0.001
Platelet, × 10^3^/mm^3^	234.0 ± 72.4	231.9 ± 71.4	238.9 ± 74.8	< 0.001
Total cholesterol, mg/dL	174.8 ± 44.1	171.0 ± 43.2	183.9 ± 45.0	< 0.001
Triglyceride, mg/dL	143.2 ± 98.2	145.1 ± 97.0	138.5 ± 101.1	0.002
HDL cholesterol, mg/dL	43.9 ± 11.8	43.9 ± 11.8	43.7 ± 11.7	0.327
LDL cholesterol, mg/dL	106.2 ± 37.8	102.0 ± 36.4	116.5 ± 39.1	< 0.001
Glucose, mg/dL	133.7 ± 57.4	127.3 ± 51.0	151.8 ± 69.6	< 0.001
Creatinine, mg/dL	1.06 ± 0.96	1.06 ± 0.96	1.07 ± 0.95	0.423
PRU	217.6 ± 78.7	217.9 ± 76.8	216.8 ± 83.0	0.515
HPR	3653 (34.0)	2561 (33.7)	1092 (34.8)	0.266
AIP, unit	0.46 ± 0.30	0.46 ± 0.29	0.44 ± 0.30	< 0.001
High AIP	3981 (37.1)	2878 (37.9)	1103 (35.2)	0.008
Medication at discharge				
Beta blocker	6205 (57.8)	3875 (51.0)	2330 (74.3)	< 0.001
Angiotensin blockade	6316 (58.8)	4113 (54.1)	2203 (70.2)	< 0.001
Calcium channel blocker	2581 (24.0)	2094 (27.6)	487 (15.5)	< 0.001
Statin	9522 (88.7)	6696 (88.1)	2826 (90.1)	0.003

Values are given as mean (SD) or absolute number (%).

*AIP* atherogenic index of plasma; *AMI* acute myocardial infarction; *BMI* body mass index; *CVA* cerebrovascular accident; *CTO* chronic total occlusion; *DES* drug-eluting stent; *HDL* high-density lipoprotein; *HPR* high platelet reactivity; *LDL* low-density lipoprotein; *LVEF* left ventricular ejection fraction; *PCI* percutaneous coronary intervention; *PRU* P2Y12 reaction unit.

### Association of AIP with the risk of HPR according to AMI

**Figure 1:**
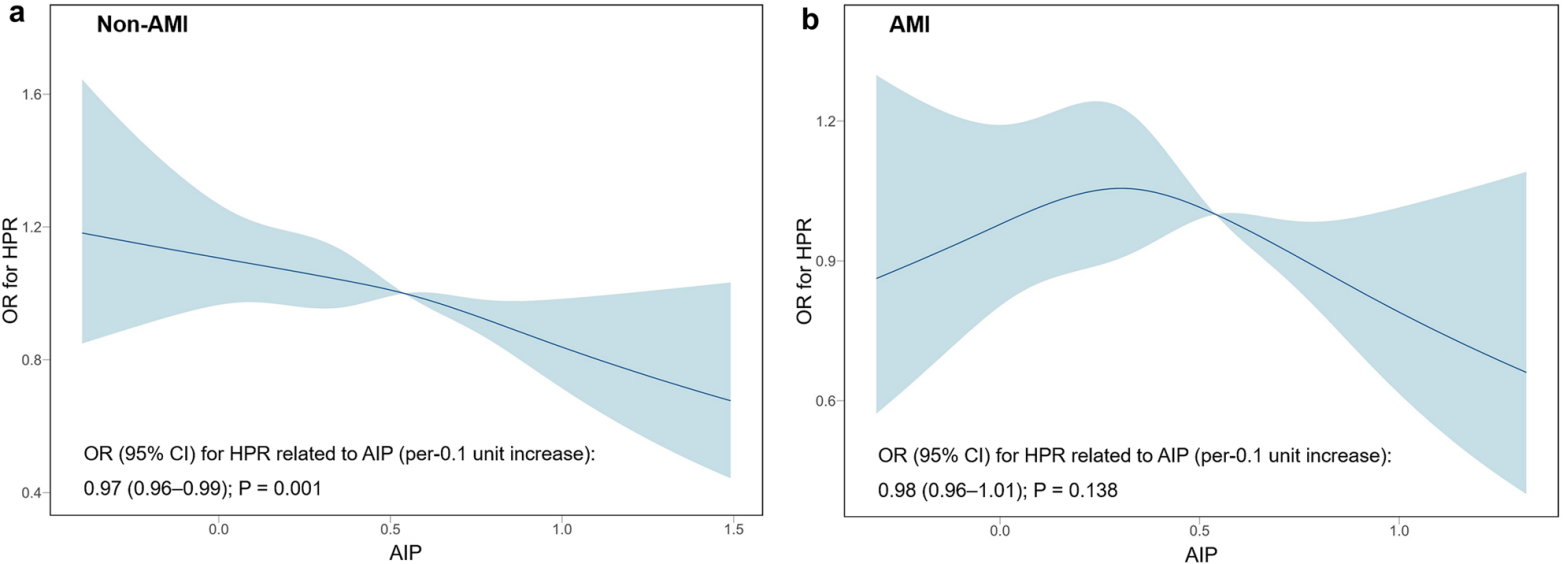
Restricted cubic spline analysis for the association of AIP levels with the risk of HPR. The reference AIP for the analysis is 0.54.

The results of the restricted cubic spine analysis for the association of AIP with the risk of HPR according to AMI status are presented in Fig. 1. With increasing AIP levels (per-0.1 unit), the risk of HPR was decreased in patients with non-AMI (OR 0.97, 95% CI 0.96–0.99; P = 0.001). However, no significant association between AIP and the risk of HPR was observed in patients with AMI (OR 0.98, 95% CI 0.96–1.01; P = 0.138). Regarding the association between high AIP and the risk of HPR according to AMI status, high AIP was significantly associated with the decreased risk of HPR in patients with non-AMI (OR 0.85, 95% CI 0.77–0.94; P = 0.002), but not in patients with AMI (OR 0.90, 95% CI 0.77–1.05; P = 0.180). These associations between high AIP and HPR risk were consistently observed after adjusting for clinical variables (Table 2).

**Table 2:** Association between high AIP and the risk of HPR according to the presentation of AMI.

	Non-AMI (n = 7599)		AMI (n = 3136)	
	OR (95% CI)	P	OR (95% CI)	P
Model 1	0.85 (0.77–0.94)	0.002	0.90 (0.77–1.05)	0.180
Model 2	0.89 (0.80–0.99)	0.030	0.99 (0.83–1.18)	0.918
Model 3	0.89 (0.79–0.99)	0.028	1.00 (0.84–1.19)	0.986

*AIP* atherogenic index of plasma; *AMI* acute myocardial infarction; *BMI* body mass index; *CI* confidence interval; *CTO* chronic total occlusion; *HPR* high platelet reactivity; *OR* odds ratio.

Model 1: unadjusted.

Model 2: adjusted for age ≥ 75 years, sex, hypertension, diabetes, dyslipidemia, obesity, smoking, and chronic kidney disease.

Model 3: Model 2 + adjusted for medical therapy, including beta-blockers, angiotensin blockers, calcium channel blockers, and statins.

### Adverse clinical outcomes

**Figure 2:**
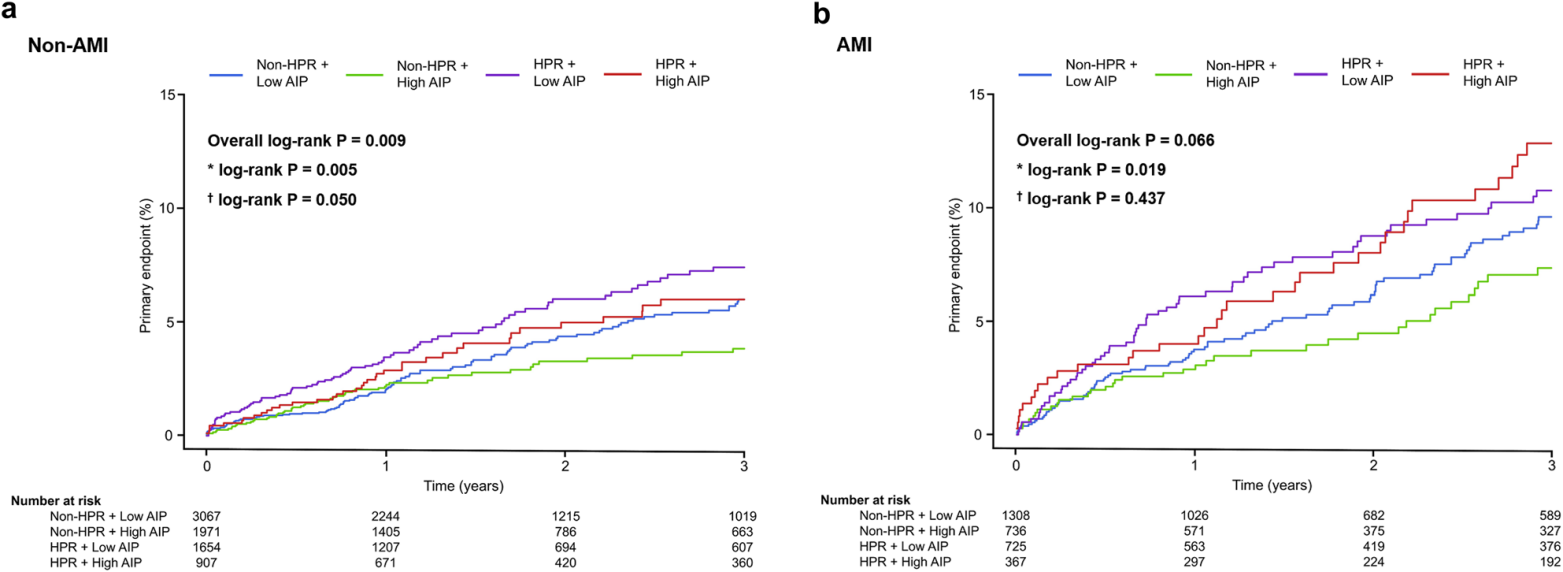
Three-year cumulative incidence of the primary endpoint. The cumulative incidence of the primary endpoint is shown according to HPR and AIP status. Asterisk: log-rank P for non-HPR vs. HPR; dagger: log-rank P for low AIP vs. high AIP.

The 3-year adverse clinical outcomes are shown in Table 3. Patients without HPR had a lower occurrence of composite outcomes than those with HPR for both non-AMI (3.2% vs. 4.7%, P = 0.002) and AMI (6.0% vs. 8.7%, P = 0.005). However, compared to patients with low AIP, those with high AIP tended to have a lower incidence of composite outcomes in non-AMI patients (4.0% vs. 3.2%; P = 0.058), which was statistically not significant. No difference in the occurrence of composite outcomes according to low and high AIP status was observed in participants with AMI (6.4% vs. 7.2%; P = 0.404). Regarding the individual components of composite outcomes, patients without HPR showed a lower occurrence of all-cause death than those with HPR in both non-AMI (1.6% vs. 2.9%; P < 0.001) and AMI (3.1% vs. 5.0%; P = 0.006). The occurrence of MI was significantly higher in participants with low AIP than in those with high AIP among patients with non-AMI (1.0% vs. 0.5%; P = 0.010); however, no difference in the occurrence of MI between low and high AIP statuses was observed in patients with AMI (2.3% vs. 2.1%; P = 0.682). The occurrence of CVA and major bleeding did not differ according to the HPR and AIP status in both non-AMI and AMI patients. The results of the Kaplan–Meier survival analysis for the cumulative incidence of the primary endpoint according to the HPR and AIP status in non-AMI and AMI participants are presented in Fig. 2.

**Table 3:** Adverse clinical events related to HPR and AIP status.

	Non-HPR + low AIP	Non-HPR + high AIP	HPR + low AIP	HPR + high AIP	P
Non-AMI (n = 7599)	n = 3067	n = 1971	n = 1654	n = 907	
Primary endpoint*	109 (3.6)	54 (2.7)	82 (5.0)	38 (4.2)	0.004
All-cause death*	54 (1.8)	27 (1.4)	48 (2.9)	27 (3.0)	0.001
MI^†^	31 (1.0)	8 (0.4)	18 (1.1)	6 (0.7)	0.066
CVA	31 (1.0)	21 (1.1)	24 (1.5)	8 (0.9)	0.475
Major bleeding	15 (0.5)	12 (0.6)	10 (0.6)	2 (0.2)	0.536
AMI (n = 3136)	n = 1308	n = 736	n = 725	n = 367	
Primary endpoint*	86 (6.6)	37 (5.0)	61 (8.4)	34 (9.3)	0.019
All-cause death*	45 (3.4)	18 (2.4)	38 (5.2)	17 (4.6)	0.029
MI	30 (2.3)	11 (1.5)	17 (2.3)	12 (3.3)	0.295
CVA	19 (1.5)	10 (1.4)	9 (1.2)	7 (1.9)	0.848
Major bleeding	8 (0.6)	9 (1.2)	8 (1.1)	3 (0.8)	0.492

Values are given as absolute number (%).

*AIP* atherogenic index of plasma; *AMI* acute myocardial infarction; *CVA* cerebrovascular accident; *HPR* high platelet reactivity; *MI* myocardial infarction.

*P < 0.05, between non-HPR and HPR; ^†^P < 0.05 between low and high AIP.

### Clinical variables and the risk of primary endpoint

Age, hypertension, diabetes, chronic kidney disease, and HPR were significantly and positively associated with the risk of the primary endpoint irrespective of AMI status. Unlike other traditional risk factors, obesity was inversely associated with the risk of the primary endpoint in both patients with non-AMI and AMI (Table 4). The results regarding the association between AIP (per-0.1 unit increase) and the risk of the primary endpoint showed that an increase of AIP was inversely associated with the risk of primary endpoint in patients with non-AMI (HR 0.94, 95% CI 0.90–0.98; P = 0.002), not in patients with AMI (HR 0.98, 95% CI 0.93–1.02; P = 0.254) in unadjusted model. Among patients with non-AMI, this association of AIP with the risk of the primary endpoint was consistently observed after consecutive adjustment of age ≥ 75 years, sex, hypertension, diabetes, dyslipidemia, obesity, smoking, chronic kidney disease, the use of beta blocker, angiotensin blockade, calcium channel blocker and statin, HPR, multivessel disease, bifurcation lesion, chronic total occlusion (CTO) lesion, number of stents, total length of stents, and minimum diameter of stents; however, a significant association of AIP with the risk of primary endpoint was not identified after adjusting for LVEF (Table 5).

**Table 4:** Association of clinical variables with the risk of primary endpoint.

	Non-AMI (n = 7599)		AMI (n = 3136)	
	HR (95% CI)	P	HR (95% CI)	P
Age, year	1.05 (1.04–1.07)	< 0.001	1.05 (1.03–1.06)	< 0.001
Male	1.11 (0.87–1.43)	0.398	0.81 (0.61–1.07)	0.136
Hypertension	1.54 (1.19–2.00)	0.001	1.57 (1.19–2.06)	0.001
Diabetes	1.32 (1.05–1.67)	0.019	1.42 (1.08–1.86)	0.013
Dyslipidemia	1.13 (0.88–1.45)	0.342	0.98 (0.74–1.30)	0.906
Obesity	0.55 (0.43–0.71)	< 0.001	0.51 (0.37–0.70)	< 0.001
Smoking	0.73 (0.54–0.98)	0.038	0.77 (0.58–1.02)	0.065
Chronic kidney disease	2.30 (1.81–2.92)	< 0.001	2.28 (1.74–2.99)	< 0.001
HF	2.73 (1.80–4.13)	< 0.001	3.84 (2.75–5.35)	< 0.001
HPR	1.40 (1.11–1.78)	0.005	1.38 (1.05–1.80)	0.019
High AIP	0.78 (0.61–1.00)	0.050	0.89 (0.67–1.19)	0.437
Multivessel disease	1.11 (0.88–1.41)	0.373	1.53 (1.17–2.01)	0.002
Bifurcation lesion	1.13 (0.83–1.56)	0.433	1.15 (0.80–1.64)	0.451
CTO lesion	1.13 (0.75–1.70)	0.565	1.71 (1.08–2.70)	0.023
Number of stents	1.09 (0.95–1.25)	0.209	1.14 (0.97–1.35)	0.113
Total length of stent, mm	1.00 (0.99–1.01)	0.104	1.00 (0.99–1.01)	0.149
Minimal diameter of stent, mm	0.63 (0.47–0.84)	0.001	0.87 (0.65–1.15)	0.321
Beta blocker	1.23 (0.97–1.56)	0.082	0.83 (0.62–1.11)	0.213
Angiotensin blockade	1.15 (0.91–1.46)	0.244	1.18 (0.87–1.59)	0.293
Calcium channel blocker	1.21 (0.94–1.56)	0.148	0.99 (0.69–1.41)	0.946
Statin	1.21 (0.84–1.74)	0.309	0.94 (0.62–1.44)	0.789

HF was defined as a reduced LVEF less than 40%

*AIP* atherogenic index of plasma; *AMI* acute myocardial infarction; *BMI* body mass index; *CI* confidence interval; *CTO* chronic total occlusion; *HF* heart failure; *HPR* high platelet reactivity; *HR* hazard ratio; *LVEF* left ventricular ejection fraction.

**Table 5:** Association of AIP (per-0.1 unit increase) with the risk of primary endpoint according to the presentation of AMI.

	Non-AMI (n = 7599)		AMI (n = 3136)	
	HR (95% CI)	P	HR (95% CI)	P
Model 1	0.94 (0.90–0.98)	0.002	0.98 (0.93–1.02)	0.254
Model 2	0.94 (0.90–0.98)	0.004	1.00 (0.95–1.05)	0.967
Model 3	0.94 (0.90–0.98)	0.003	1.00 (0.95–1.06)	0.883
Model 4	0.94 (0.90–0.98)	0.004	1.00 (0.95–1.06)	0.869
Model 5	0.96 (0.92–1.00)	0.076	1.01 (0.96–1.07)	0.702

*AIP* atherogenic index of plasma; *AMI* acute myocardial infarction; *BMI* body mass index; *CI* confidence interval; *CTO* chronic total occlusion; *HPR* high platelet reactivity; *HR* hazard ratio; *LVEF* left ventricular ejection fraction.

The adjusted variables for Models 1, 2, and 3 are the same as those in Table 2.

Model 4: Model 3 + adjusted for HPR, multivessel disease, bifurcation lesions, CTO lesions, number of stents, total stent length, and minimum stent diameter.

Model 5: Modle 4 + adjusted for LVEF.

## Discussion

In this prespecified analysis of the PTRG-DES consortium, patients with HPR showed a higher cumulative rate of the primary endpoint than those without HPR, irrespective of the presentation of AMI. The major findings of the present study were that (1) AIP levels were inversely associated with the risk of HPR in only non-AMI patients and (2) AIP levels did not show an independent prognostic value in either non-AMI or AMI patients who underwent successful PCI with DES.

The significance of triglyceride in the primary prevention of atherosclerotic CV disease has recently been emphasized in clinical practice^16,17^. Elevated serum triglyceride levels stimulate the activity of cholesteryl ester transfer proteins, which exchange triglycerides from triglyceride-rich lipoproteins with cholesteryl esters from HDL and LDL^18^. Triglyceride enrichment of HDL and LDL particles makes them better substrates for lipolysis, leading to HDL catabolism and elimination, and the formation of denser LDL particles. Recent large cohort data demonstrated that increased HDL cholesterol levels were closely related to a lower risk of obstructive CAD, especially in non-diabetic patients who achieved optimal LDL cholesterol levels^19^. Considering the complex interactions in lipoprotein metabolism, AIP, which is based on the ratio of triglycerides to HDL-C, has been suggested as an effective marker of plasma atherogenicity^3,4^. Although previous studies have reported a strong relationship between AIP and subclinical coronary atherosclerosis^5,20^, little is known about the association of AIP with platelet reactivity and prognosis in the contemporary era of PCI with DES.

To the best of our knowledge, this is the first study which evaluated the association between plasma atherogenicity marker and platelet reactivity according to the presentation of AMI. In this PTRG-DES consortium study, higher AIP levels were associated with a lower risk of HPR in patients without AMI. This suggests that the presence of phenomenon maintaining a balance between plasma atherogenicity and platelet reactivity may play a substantial role in the development of AMI. Both increased triglycerides and decreased HDL cholesterol levels are typical types of dyslipidemia in the obese population. Numerous previous studies reported that an increase in BMI is associated with improved short- and long-term prognosis, which is called the phenomenon of “obesity paradox” or “reverse epidemiology,” in patients with and without AMI^21–23^. In the present study, we also observed (1) a favorable effect of obesity on the risk of the primary endpoint and (2) a positive relationship between BMI and AIP levels (Supplementary Fig. 1). Alike the clinical features of obese patients in previous studies which reported the obesity paradox in the era of PCI with DES^23,24^, the present study found that patients with high AIP were significantly younger (62.3 ± 11.2 vs. 65.6 ± 10.5 years; P < 0.001) and tended to have a lower prevalence of heart failure with LVEF < 40% (4.5% vs. 6.1%; P = 0.002) compared with those with low AIP among overall participants. These facts might influence on the favorable effect of high AIP on the risk of primary endpoint in the unadjusted statistical model of present study.

It is well-established that HPR has an independent prognostic value after PCI with DES^6–8^. Regarding the association of HPR with the risk of primary endpoint, the present study found that the prognostic value of HPR was significantly improved with consideration of clinical risk factors and heart failure together irrespective of the presentation of AMI; however, further adjustment of high AIP could not improve the prognostic value of HPR in both non-AMI and AMI patients (Supplementary Table 2). According to the results from the PROMINENT (Pemafibrate to Reduce Cardiovascular Outcomes by Reducing Triglycerides in Patients with Diabetes) trial which was performed in patients with type 2 diabetes, mild-to-moderate hypertriglyceridemia, and low HDL and LDL cholesterol levels, the primary endpoint of non-fatal MI, ischemic stroke, coronary revascularization, or death from cardiovascular causes was not lower among patients who received pemafibrate than among those who received placebo^25^. Similarly, previous studies have shown no beneficial effects of increased HDL levels on adverse clinical outcomes, especially in patients with established CV disease or those at high risk^26–28^. These findings might show a limited role of AIP as an independent prognostic marker in the field of secondary prevention. Further randomized investigations regarding the prognostic value of AIP with stricter measures to control LDL cholesterol levels are necessary in the recent era of PCI with DES^29^.

The present study has several limitations. First, this was a post-hoc analysis of a non-randomized observational cohort registry. Thus, selection bias may have affected the results of the study. Second, we only observed the association of AIP with HPR and were not able to confirm their causal relationship because of the retrospective nature of current study. Third, serial evaluations of AIP and platelet function were not performed during the follow-up period. Finally, this study included only an East Asian population, which may limit its generalizability. However, the PTRG-DES consortium is the largest registry for evaluating platelet function and long-term prognosis in the era of PCI with DES. The current study is unique in that different associations between AIP and the risk of HPR according to the presentation of AMI were identified among East Asians after successful PCI with DES.

In summary, an inverse association between plasma atherogenicity assessed by AIP and the risk of HPR was observed in non-AMI patients; this association was consistently observed in these patients after adjusting for numerous clinical and procedural factors. Among participants of PTRG-DES who underwent successful PCI using DES, the AIP did not show an independent prognostic value irrespective of the presentation of AMI. The results of the present study suggest that the association between plasma atherogenicity and platelet reactivity plays an important role in AMI development. Further clinical investigations are required to confirm the results of this study.

### Supplementary Information

Supplementary Information.

## Data Availability

The datasets used and analyzed in the current study are available from the corresponding author upon reasonable request.
