# Supplementary material for: Different association of atherogenic index of plasma with the risk of high platelet reactivity according to the presentation of acute myocardial infarction
Source: Sci Rep. 2024 May 13;14:10894. doi: 10.1038/s41598-024-60999-3 (PMC11091133; doi:10.1038/s41598-024-60999-3)

Different association of atherogenic index of plasma with the risk of high platelet reactivity according to the presentation of acute myocardial infarction

Running title:

Association between AIP and HPR according to AMI

Authors:

Ki-Bum Won[1], Hyeon Jeong Kim[2], Jun Hwan Cho[1], Sang Yup Lee[1], Ae-Young Her[3], Byeong-Keuk Kim[4], Hyung Joon Joo[5], Yongwhi Park[6], Kiyuk Chang[7], Young Bin Song[8], Sung Gyun Ahn[9], Jung-Won Suh[10], Jung Rae Cho[11], Hyo-Soo Kim[12], Moo Hyun Kim[13], Do-Sun Lim[5], Sang-Wook Kim[1], Young-Hoon Jeong[1], and Eun-Seok Shin[14]*

E-mail:

Ki-Bum Won: kbwon99@naver.com

Hyeon Jeong Kim: aneace.1@gmail.com

Jun Hwan Cho: cardio.jhcho@gmail.com

Sang Yup Lee: louisahj@gmail.com

Ae-Young Her: hermartha1@gmail.com

Byeong-Keuk Kim: kimbk@yuhs.ac

Hyung Joon Joo: drjoohj@gmail.com

Yongwhi Park: angio2000@hanmail.net

Kiyuk Chang: kiyuk@catholic.ac.kr

Young Bin Song: youngbin.song@gmail.com

Sung Gyun Ahn: sgahn@yonsei.ac.kr

Jung-Won Suh: suhjw1@gmail.com

Jung Rae Cho: jrjoe@naver.com

Hyo-Soo Kim: hyosoo@snu.ac.kr

Moo Hyun Kim: kimmh@dau.ac.kr

Do-Sun Lim: dslmd@kumc.or.kr

Sang-Wook Kim: swkimcv@cau.ac.kr

Young-Hoon Jeong: goodoctor@naver.com

Eun-Seok Shin: sesim1989@gmail.com

Affiliations:

[1]Division of Cardiology, Chung-Ang University Gwangmyeong Medical Center, Chung-Ang University College of Medicine, Gwangmyeong, South Korea

[2]Division of Cardiology, Busan Veterance Hospital, Busan, South Korea

[3]Division of Cardiology, Kangwon National University School of Medicine, Chuncheon, South Korea.

[4]Division of Cardiology, Severance Cardiovascular Hospital, Yonsei University College of Medicine, Seoul, South Korea

[5]Division of Cardiology, Cardiovascular Center, Korea University Anam Hospital, Korea University College of Medicine, Seoul, South Korea

[6]Division of Cardiology, Gyeongsang National University Changwon Hospital, Gyeongsang National University School of Medicine, Changwon, South Korea

[7]Division of Cardiology, Seoul St. Mary's Hospital, College of Medicine, Catholic University of Korea, Seoul, South Korea

[8]Division of Cardiology, Samsung Medical Center, Sungkyunkwan University School of Medicine, Seoul, South Korea

[9]Division of Cardiology, Yonsei University Wonju Severance Christian Hospital, Wonju, South Korea

[10]Division of Cardiology, Seoul National University Bundang Hospital, Seongnam, South Korea

[11]Division of Cardiology, Kangnam Sacred Heart Hospital, Hallym University College of Medicine, Seoul, South Korea

[12]Division of Cardiology, Seoul National University Hospital, Seoul, South Korea

[13]Division of Cardiology, Dong-A University Hospital, Busan, South Korea

[14]Division of Cardiology, Ulsan University Hospital, University of Ulsan College of Medicine, Ulsan, South Korea

***Address for correspondence:**

Eun-Seok Shin, MD, PhD

Division of Cardiology, Ulsan University Hospital, University of Ulsan College of Medicine,

877 Bangeojinsunhwando-ro, Dong-gu, Ulsan, 44033, South Korea

Email: sesim1989@gmail.com

Supplementary table 1. Baseline characteristics according to HPR and AIP status in non-AMI and AMI patients

	Non-HPR + Low AIP	Non-HPR + High AIP	HPR + Low AIP	HPR + High AIP	P
Non-AMI (n = 7599)	n = 3067	n = 1971	n = 1654	n = 907	
Age, years	64.3 ± 10.0	61.5 ± 10.4	67.6 ± 9.5	65.8 ± 9.8	<0.001
Male	2181 (71.1)	1490 (75.6)	899 (54.4)	490 (54.0)	<0.001
LVEF, %	60.8 ± 10.2	60.9 ± 9.6	60.7 ± 10.1	61.3 ± 9.2	0.579
Hypertension	1838 (59.9)	1222 (62.0)	1148 (69.4)	617 (68.0)	<0.001
Diabetes mellitus	996 (32.5)	758 (38.5)	605 (36.6)	413 (45.5)	<0.001
Dyslipidemia	1682 (54.8)	1727 (87.6)	913 (55.2)	789 (87.0)	<0.001
Obesity	1211 (39.5)	1077 (54.6)	680 (41.1)	484 (53.4)	<0.001
Smoking	717 (23.4)	646 (32.8)	246 (14.9)	182 (20.1)	<0.001
Chronic kidney disease	489 (15.9)	377 (19.1)	389 (23.5)	254 (28.0)	<0.001
Peripheral artery disease	290 (9.5)	197 (10.0)	193 (11.7)	122 (13.5)	0.002
Previous PCI	482 (15.7)	286 (14.5)	254 (15.4)	129 (14.2)	0.562
Previous CVA	224 (7.3)	126 (6.4)	119 (7.2)	57 (6.3)	0.510
Multivessel disease	1067 (34.8)	760 (38.6)	591 (35.7)	394 (43.4)	<0.001
Bifurcation lesion	343 (11.2)	221 (11.2)	189 (11.4)	115 (12.7)	0.643
CTO lesion	228 (7.4)	170 (8.6)	106 (6.4)	53 (5.8)	0.019
Number of stents	1.6 ± 0.8	1.6 ± 0.8	1.6 ± 0.8	1.7 ± 0.8	0.151
Length of stent, mm	35.5 ± 22.9	36.1 ± 23.2	34.8 ± 22.9	37.5 ± 23.9	0.031
Minimal diameter of stent, mm	2.99 ± 0.43	3.02 ± 0.44	2.99 ± 0.41	2.97 ± 0.41	0.014
Beta blocker	1513 (49.3)	1027 (52.1)	856 (51.8)	479 (52.8)	0.114
Angiotensin blockade	1617 (52.7)	1076 (54.6)	918 (55.5)	502 (55.3)	0.220

Calcium channel blocker	826 (26.9)	558 (28.3)	471 (28.5)	239 (26.4)	0.471
Statin	2669 (87.0)	1764 (89.5)	1461 (88.3)	802 (88.4)	0.064
AMI (n = 3136)	n = 1308	n = 736	n = 725	n = 367	
Age, years	64.4 ± 11.9	59.0 ± 12.9	68.5 ± 11.0	64.8 ± 12.1	<0.001
Male	951 (72.7)	590 (80.2)	416 (57.4)	235 (64.0)	<0.001
LVEF, %	54.2 ± 10.6	55.7 ± 9.5	53.2 ± 11.3	54.7 ± 10.4	<0.001
Hypertension	664 (50.8)	369 (50.1)	412 (56.8)	207 (56.4)	0.012
Diabetes mellitus	348 (26.6)	207 (28.1)	225 (31.0)	162 (44.1)	<0.001
Dyslipidemia	726 (55.5)	680 (92.4)	404 (55.7)	333 (90.7)	<0.001
Obesity	404 (30.9)	362 (49.2)	188 (25.9)	144 (39.2)	<0.001
Smoking	511 (39.1)	353 (48.0)	212 (29.2)	154 (42.0)	<0.001
Chronic kidney disease	259 (19.8)	123 (16.7)	207 (28.6)	126 (34.3)	<0.001
Peripheral artery disease	194 (14.8)	113 (15.4)	134 (18.5)	62 (16.9)	0.166
Previous PCI	127 (9.7)	61 (8.3)	75 (10.3)	31 (8.4)	0.499
Previous CVA	78 (6.0)	43 (5.8)	65 (9.0)	33 (9.0)	0.017
Multivessel disease	572 (43.7)	321 (43.6)	374 (51.6)	165 (45.0)	0.004
Bifurcation lesion	156 (11.9)	74 (10.1)	115 (15.9)	45 (12.3)	0.007
CTO lesion	80 (6.1)	40 (5.4)	42 (5.8)	28 (7.6)	0.534
Number of stents	1.6 ± 0.8	1.6 ± 0.7	1.6 ± 0.8	1.6 ± 0.7	0.388
Length of stent, mm	35.7 ± 21.0	36.4 ± 21.6	37.3 ± 22.5	36.2 ± 21.4	0.459
Minimal diameter of stent, mm	3.04 ± 0.45	3.13 ± 0.49	3.03 ± 0.46	3.07 ± 0.47	<0.001
Beta blocker	949 (72.6)	581 (78.9)	537 (74.1)	263 (71.7)	0.008
Angiotensin blockade	889 (68.0)	521 (70.8)	536 (73.9)	257 (70.0)	0.044
Calcium channel blocker	201 (15.4)	111 (15.1)	116 (16.0)	59 (16.1)	0.951
Statin	1173 (89.7)	683 (92.8)	648 (89.4)	322 (87.7)	0.029

Values are given as absolute number (%).

AIP atherogenic index of plasma; *AMI* acute myocardial infarction; *CVA* cerebrovascular accident; *HPR* high platelet reactivity; *MI* myocardial infarction

Supplementary table 2. C statics for evaluating the risk of primary endpoint regarding HPR alone and consecutively added models of clinical risk factors, HF, and AIP in non-AMI and AMI patients

Model	C-statics	P compared with model 1 (NRI P/ IDI P)	P compared with model 2 (NRI P/ IDI P)	P compared with model 3 (NRI P/ IDI P)
Non-AMI				
Model 1: HPR only	0.545	–	–	–
Model 2: Model 1 + clinical risk factors of age, hypertension, diabetes, obesity, smoking, and chronic kidney disease	0.691	<0.001 (<0.001/<0.001)	–	–
Model 3: Model 2 + HF	0.702	<0.001 (<0.001/<0.001)	0.059 (0.065/0.005)	–
Model 4: Model 3 + high AIP	0.702	<0.001 (<0.001/<0.001)	0.068 (0.136/0.004)	0.760 (0.235/0.718)
AMI				
Model 1: HPR only	0.547	–	–	–
Model 2: Model 1 + clinical risk factors of age, hypertension, diabetes, obesity, smoking, and chronic kidney disease	0.685	<0.001 (<0.001/<0.001)	–	–
Model 3: Model 2 + HF	0.705	<0.001 (<0.001/<0.001)	0.034 (0.053/<0.001)	–
Model 4: Model 3 + high AIP	0.705	<0.001	0.045	0.958

		(<0.001/<0.001)	(0.059/<0.001)	(0.571/0.226)

HF was defined as a reduced LVEF less than 40%.

AIP atherogenic index of plasma; *AMI* acute myocardial infarction; *HF* heart failure; *HPR* high platelet reactivity; *IDI* integrated discrimination index; *LVEF* left ventricular ejection fraction; *NRI* net reclassification index

Supplementary figure 1. Association between BMI and AIP in non-AMI and AMI patients

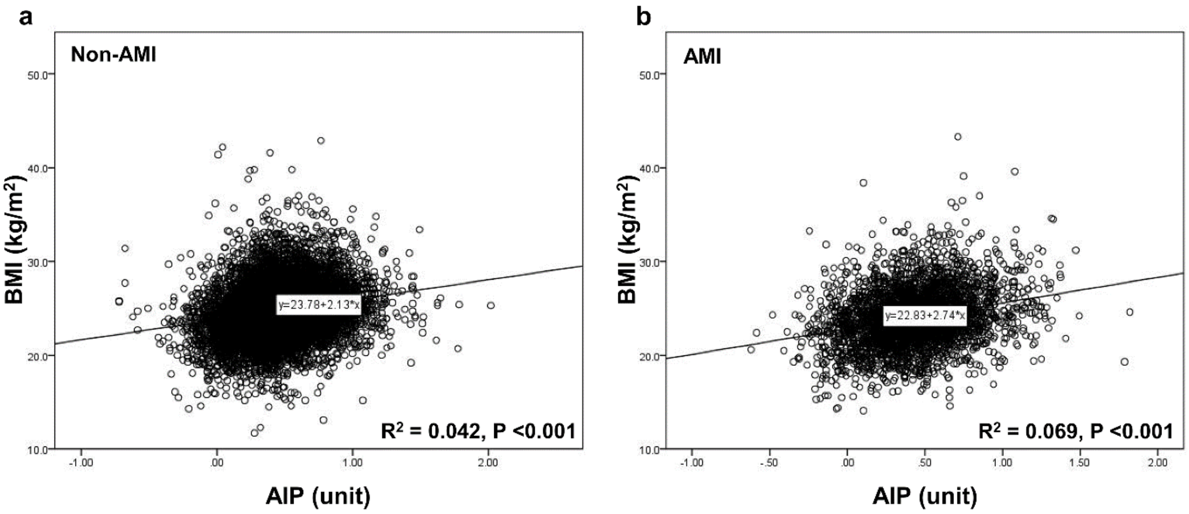

Supplement: Supplementary file 1 — Supplementary Information. [file 41598_2024_60999_MOESM1_ESM.pdf]
